# Follistatin supplementation induces changes in CDX2 CpG methylation and improves in vitro development of bovine SCNT preimplantation embryos

**DOI:** 10.1186/s12958-021-00829-7

**Published:** 2021-09-13

**Authors:** Mohamed Ashry, Chunyan Yang, Sandeep K. Rajput, Joseph K. Folger, Jason G. Knott, George W. Smith

**Affiliations:** 1 Laboratory of Mammalian Reproductive Biology and Genomics, Department of Animal Science, Reproductive and Developmental Sciences Program, Michigan State University, East Lansing, MI USA; 2 Developmental Epigenetics Laboratory, Department of Animal Science, Reproductive and Developmental Sciences Program, Michigan State University, East Lansing, MI USA; 3 Department of Theriogenology, Faculty of Veterinary Medicine, Cairo University, Giza, Egypt; 4 Guangxi Buffalo Research Institute, Chinese Academy of Agricultural Science, Nanning, China

**Keywords:** Bovine preimplantation embryos, SCNT, *CDX2*, DNA methylation, Follistatin

## Abstract

Caudal Type Homeobox 2 (CDX2) is a key regulator of trophectoderm formation and maintenance in preimplantation embryos. We previously demonstrated that supplementation of exogenous follistatin, during in vitro culture of bovine IVF embryos, upregulates *CDX2* expression, possibly, via alteration of the methylation status of *CDX2* gene. Here, we further investigated the effects of exogenous follistatin supplementation on developmental competence and *CDX2* methylation in bovine somatic cell nuclear transfer (SCNT) embryos. SCNT embryos were cultured with or without follistatin for 72h, then transferred into follistatin free media until d7 when blastocysts were collected and subjected to *CDX2* gene expression and DNA methylation analysis for *CDX2* regulatory regions by bisulfite sequencing. Follistatin supplementation significantly increased both blastocyst development as well as blastocyst *CDX2* mRNA expression on d7. Three different CpG rich fragments within the *CDX2* regulatory elements; proximal promoter (fragment P1, -1644 to -1180; P2, -305 to +126) and intron 1 (fragment I, + 3030 to + 3710) were identified and selected for bisulfite sequencing analysis. This analysis showed that follistatin treatment induced differential methylation (DM) at specific CpG sites within the analyzed fragments. Follistatin treatment elicited hypomethylation at six CpG sites at positions -1374, -279, -163, -23, +122 and +3558 and hypermethylation at two CpG sites at positions -243 and +20 in promoter region and first intron of *CDX2* gene. Motif analysis using MatInspector revealed that differentially methylated CpG sites are putative binding sites for key transcription factors (TFs) known to regulate *Cdx2* expression in mouse embryos and embryonic stem cells including OCT1, AP2F, KLF and P53, or TFs that have indirect link to *CDX2* regulation including HAND and NRSF. Collectively, results of the present study together with our previous findings in IVF embryos support the hypothesis that alteration of *CDX2* methylation is one of the epigenetic mechanisms by which follistatin may regulates *CDX2* expression in preimplantation bovine embryos.

**Supplementary Information:**

The online version contains supplementary material available at 10.1186/s12958-021-00829-7.

## Introduction

Somatic cell nuclear transfer (SCNT) is a powerful tool used to propagate valuable livestock species and to generate genetically modified animals that can be used in biomedical research and therapeutic cloning [1, 2]. Despite the successful cloning of numerous species, the application of the technology is still limited due to several complications that arise from using somatic cells as nuclei donors. These difficulties include poor embryonic development, placental abnormalities, higher abortion rates, larger birth weights and perinatal mortalities [3–5]. These abnormalities are likely caused by incomplete reprogramming of the somatic cell nucleus that impact normal transcriptional programming during preimplantation development [6–8].

A previous study demonstrated that exogenous follistatin supplementation during in vitro embryo culture increased blastocyst development and trophectoderm (TE) cell number in bovine SCNT embryos [9]. Follistatin is a binding protein for specific members of the transforming growth factor β (TGF-β) super family. Follistatin protein and mRNA are upregulated in good quality oocytes [10, 11]. Numerous studies in our laboratory showed that exogenous follistatin supplementation during in vitro embryo culture improves the developmental competence of bovine preimplantation embryos produced under different conditions. These include conventional IVF [12–14], IVF using poor quality oocytes [11], and IVF using sex sorted semen [15]. In general, follistatin treatment increases blastocyst rates, *CDX2* expression and trophectoderm (TE) cell number on day 7 of development. Most recently, we demonstrated that follistatin supplementation during the initial 72h of in vitro embryo culture (IVC1) upregulated *CDX*2 expression in IVF blastocysts that was tightly associated with DNA methylation at specific CpG sites within the regulatory elements of *CDX2* gene including the proximal promoter and intron 1. These CpG sites contain putative binding sites for several key transcription factors implicated in regulating *Cdx2* expression in mouse embryos and trophoblast stem cells [16]. Importantly, increased *CDX2* expression in bovine blastocysts is associated with improved pregnancy rates after embryo transfer [17]. Therefore, the objectives of the present study were to analyze the beneficial effects of follistatin on developmental competence and *CDX2* expression in bovine SCNT embryos and to investigate whether these effects are linked to changes in DNA methylation as we previously reported in IVF embryos [16]. To address this, we cultured SCNT embryos with or without follistatin (10ng/ml) during the first 72h of culture, then at blastocyst stage, we analyzed DNA methylation at *CDX2* regulatory regions. Results show that follistatin treatment induces changes in DNA methylation at specific CpG sites within *CDX2* regulatory regions in SCNT blastocysts, these changes are associated with upregulation of *CDX2* expression.

## Materials and methods

### Chemicals and supplies

All reagents were purchased from Sigma Aldrich (Saint Louis, MO) unless mentioned otherwise.

### Oocyte collection, In vitro maturation, Enucleation, and somatic cell nuclear transfer

Bovine cumulus oocytes complexes (COCs) were retrieved from ovaries collected at local slaughterhouse in the state of Michigan. Oocyte selection and in vitro maturation (IVM) were carried out as we previously described [11, 12]. Somatic cell nuclear transfer (SCNT) was performed as reported elsewhere [18]. Briefly, in vitro matured oocytes were denuded from cumulus cells and MII oocytes with extruded first polar bodies were selected for enucleation. Bovine fibroblasts were used as donor cells and injected into the perivitelline space of each enucleated oocyte. The SCNT couplets were immediately fused in sorbitol fusion medium by a single electric pulse (2 kV/cm for 15 μs) and cultured for 0.5-1h. After fusion, the couplets were activated by 5 mM ionomycin in HH medium for 5 min then transferred to equilibrated KSOM medium containing 2 mM of 6-DMAP for 4h. Afterwards, SCNT embryos were washed and transferred into KSOM culture medium.

### In vitro embryo culture and follistatin supplementation

**Fig. 1:**
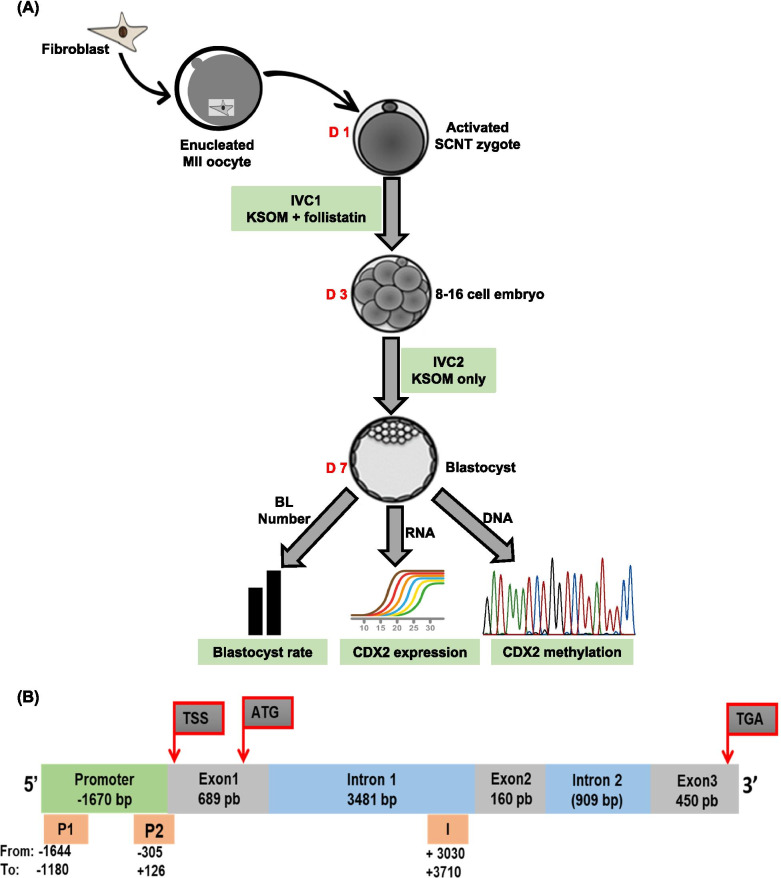
Study design and bovine *CDX2* gene structure. **A** To investigate the effects of follistatin on early development of somatic cell nuclear transfer (SCNT) embryos and *CDX2* methylation status in blastocyst, activated SCNT zygotes were cultured with or without follistatin (10 ng/ml) during IVC1, then 8-16 cell embryos were cultured in follistatin free media until day 7, when percentage of embryos developing to blastocyst stage were recorded. Then blastocysts were used for extraction of total RNA for *CDX2* gene expression analysis and genomic DNA for bisulfite sequencing analysis. **B** Schematic illustration of bovine *CDX2* gene showing the regulatory elements of the gene (proximal promoter and intron 1) and position of the fragments selected for analysis (P1, P2 and I)

The activated SCNT embryos were cultured in KSOM media with or without the maximal stimulatory dose of follistatin, 10 ng/ml (R&D Systems, Minneapolis, MN) for 72h [14]. Then, 8-16 cell embryos were cultured in follistatin free media until d7. At the end of the culture, blastocysts were collected and properly stored until used for simultaneous isolation of genomic DNA, for bisulfite sequencing and total RNA, for *CDX2* gene expression analysis. Figure 1A provides a schematic illustration of the study design.

### *CDX2* gene expression analysis

Total RNA isolation, cDNA synthesis and qRT-PCR were performed as previously described [16]. Briefly, SCNT blastocysts from control and follistatin treated groups (*n* = 15 blastocysts/group, *n* = 6 replicates) were used for isolation of genomic DNA and total RNA using Allprep DNA/RNA micro kit (Qiagen, Valencia, CA). The extracted total RNA was used for cDNA synthesis using iScript cDNA synthesis kit (BioRad, Hercules, CA) according to the manufacturer’s protocol. Quantification of the relative transcript abundance of *CDX2* gene was done by the 2^−ΔΔCt^ method as described elsewhere [19]. *CDX2* transcript abundance was normalized relative to the abundance of bovine ribosomal protein S18 (RPS18) as an endogenous control and expressed as fold change relative to the control group. Primer sequences and GenBank accession numbers were previously reported [16].

### Bisulfite sequencing analysis

Analysis of CpG content by MethPrimer software [20] revealed that the bovine *CDX2* gene (GenBank ID: NC_037339, Region 32081960-32087314) contains multiple CpG rich islands upstream and downstream of the transcription start site (TSS; +1) (genomic Position 32083604). Given the *CDX2* regulatory elements include the promoter region and intron 1 [21, 22], three different CpG rich fragments within the promoter region (fragment P1; -1644 to -1180; P2, -305 to +126) and intron 1 (fragment I + 3030 to + 3710) were selected for bisulfite-sequencing analysis. Figure 1B provides a schematic illustration of *CDX2* gene structure and position of selected fragments. Bisulfite treatment, DNA amplification, cloning and Sanger sequencing were performed following our previously reported protocols [16]. Briefly, bisulfite conversion of genomic DNA was performed using EpiTect Bisulfite Kit (Qiagen) followed by PCR amplification using GoTaq DNA Polymerase (Promega, Madison, WI). Each PCR product was extracted and purified, then cloned directly into a pCR4-TOPO vector using a TOPO-TA cloning kit for Sequencing (Invitrogen, Grand Island, NY). Cloned products were transformed into One Shot TOP10 chemically competent cells (Invitrogen). After overnight growth on selective agar plates, colony PCR was performed using M13 primers to select positive clones. Eight positive clones per replicate were selected and submitted for Sanger sequencing at Michigan State University genomic core facility. For detailed information of the bisulfite primers and different reactions conditions please see our previous publication [16].

### Analysis and presentation of bisulfite sequencing data

Raw bisulfite sequencing data were aligned to the *CDX2* reference sequence using BioEdit Sequence Alignment Editor (Ibis biosciences, Carlsbad, CA). Aligned sequences with sequence identity≥ 90 % and conversion rate ≥ 95 were analyzed by Bisulfite Sequencing DNA Methylation Analysis (BISMA) Software [23]. Individual CpG sites with ≥ 20 % methylation in either control or follistatin treated groups were analyzed by Fisher’s exact test to determine the differentially methylated CpG sites (DMS) [24, 25]. Methylation grids were generated by Methylation plotter web tool [26].

### Transcription factor motif analysis

Motif analysis was performed by MatInspector (Genomatix) to identify putative transcription factors that bind to the differentially methylated CpG sites within the regulatory regions of *CDX2* gene. A vigilant literature search was carried out to uncover any role for these transcription factors in regulating *CDX2* expression.

### Statistical analysis

Student T test was used to analyze embryo development and *CDX2* gene expression data. Arcsine transformation was applied to the percentage data before analysis. Data are presented as untransformed mean ± standard error of mean (SEM), *P* < 0.05. For DNA methylation data, after initial analysis by BISMA, individual CpG sites with ≥ 20 methylation in either control or follistatin treated groups were analyzed by Fisher’s exact test to determine the differentially methylated CpG sites.

## Results

### Follistatin supplementation improves early development and upregulates *CDX2* expression in bovine SCNT embryos

**Fig. 2:**
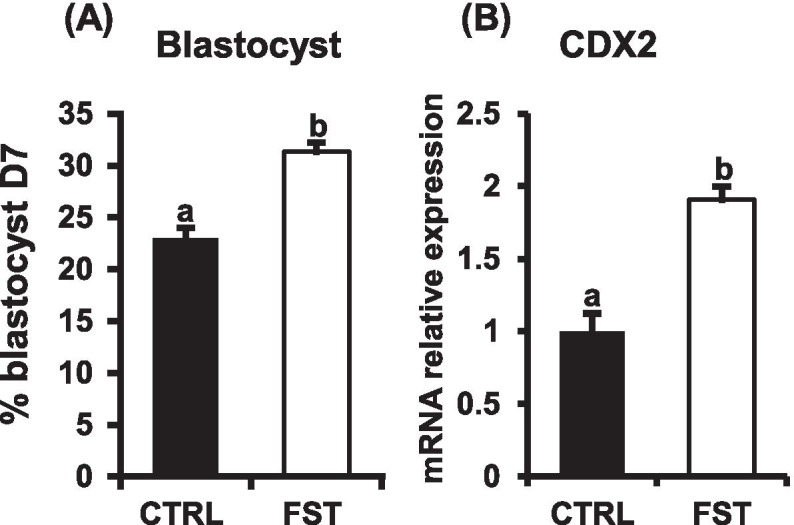
Follistatin supplementation improves blastocyst development and upregulates *CDX2* expression in bovine SCNT embryos. Activated SCNT zygotes were cultured with or without follistatin (10 ng/ml) for 72h. Then, embryos were cultured in follistatin free media until d 7. **A** Percentage of embryos that developed to the blastocyst stage. **B** QRT-PCR analysis of *CDX2* mRNA abundance in blastocysts in response to follistatin treatment. *CDX2* mRNA expression was normalized relative to the abundance of RPS18 as a housekeeping gene. Data are expressed as mean ± SEM. Values with different superscripts indicate significant differences (*P* < 0.05)

Follistatin supplementation (10ng/ml) during the initial 72h of in vitro embryo culture (IVC) triggered a significant increase in the proportion of SCNT embryos that reached the blastocyst stage at d7 (31.34 ± 0.97% Vs 20.04%± 0.85%) for follistatin and control groups, respectively (*P*<0.05; Fig. 2A). Moreover, the mRNA expression level of *CDX2* was significantly increased in d7 blastocysts derived from follistatin treated group, compared to untreated controls (*P*<0.05; Fig. 2B).

### Follistatin treatment induces changes in DNA methylation within *CDX2* gene promoter in bovine SCNT blastocysts

Bisulfite sequencing analysis was performed to investigate whether follistatin treatment (10 ng/ml), during the first 72h of IVC modified the methylation status of regulatory regions in the *CDX2* promoter in SCNT blastocysts. Therefore, we analyzed the CpG content of *CDX2* using MethPrimer software [20] (Fig. 1B). Two CpG rich fragments in the promoter region; named P1 (-1644 to -1180) and P2 (-126 to +305) were identified and selected for analysis.

**Fig. 3:**
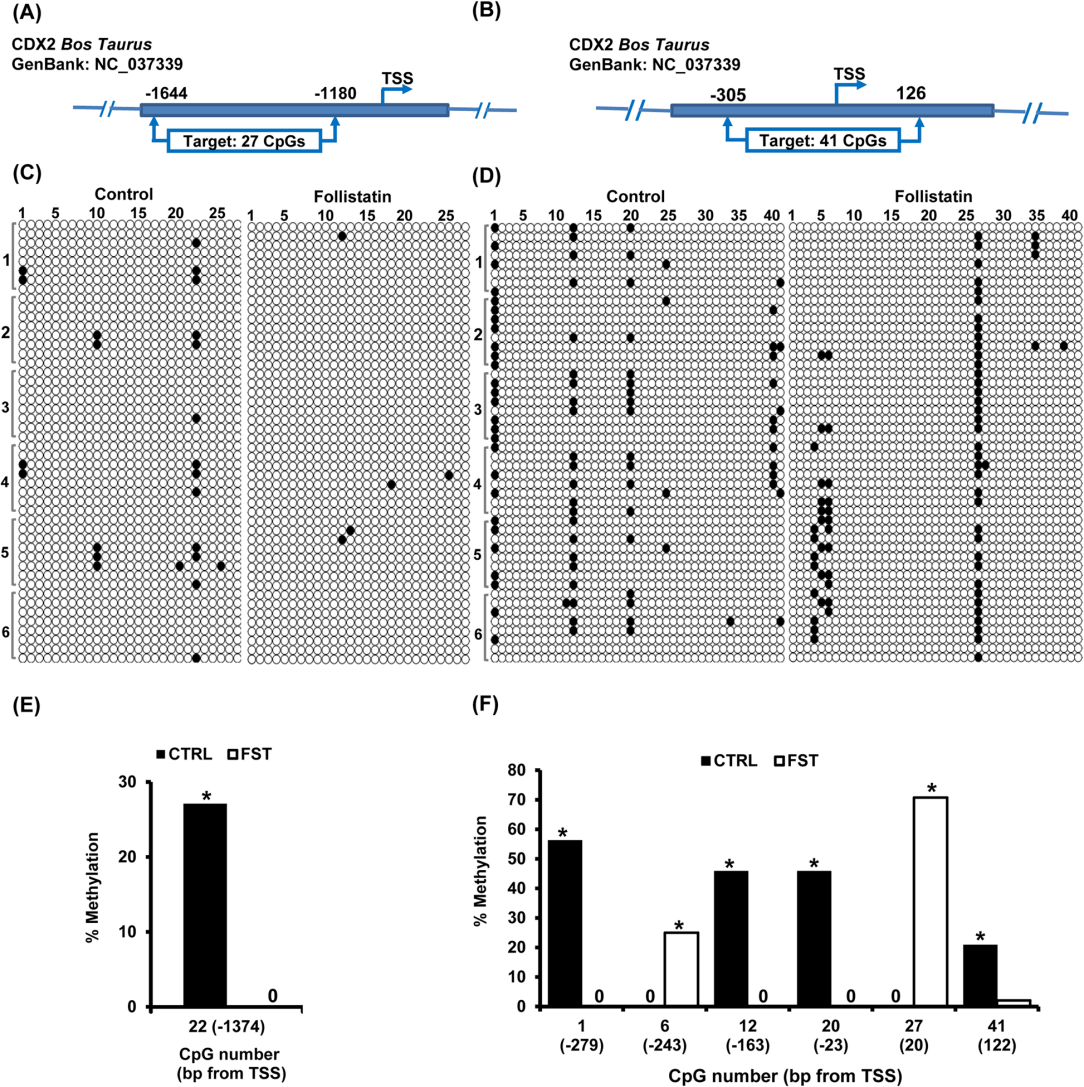
Follistatin treatment induces changes in DNA methylation within the *CDX2* gene promoter in bovine SCNT blastocysts. **A**, **B** *CDX2* gene map showing the genomic position of fragments P 1 ( -1644 to -1180) and P2(-305 to +126). **C**, **D** Individual CpG methylation profile in fragments P1 and P2, genomic DNA, from untreated control and follistatin treated groups (*n*= 6 replicates, *n*= 15 blastocysts/group), was subjected to bisulfite treatment followed by PCR amplification, cloning, transformation and finally colony PCR to select positive clones (*n*= 8 clones/replicate) for Sanger sequencing. In the methylation grid, rows represent the sequenced clones (Clones are arranged according to their replicates, replicate number are shown in the left) and columns represent CpG sites. Unmethylated CpG sites are represented by open circles while methylated CpG sites are represented by closed circle. **E**, **F** Individual CpG sites with ≥ 20% methylation, at least in one group. Values show the methylation percentage of each CpG site and those values marked with an asterisk are significantly different (*P < 0.05)

For fragment P1, the target sequence was 464 bp in length (-1644 to -1180) and included 27 CpG sites (Fig. 3A). The overall methylation status of this fragment was 1.9 ± 0.45 % and 0.4 ± 0.16% for control and follistatin treated groups, respectively (Fig. 3C). Analysis of individual CpG sites, revealed that eight CpG sites were methylated in 2.1 - 27.1 % of the sequenced clones (Fig. 3C). Further analysis by Fisher’s exact test, to determine the differentially methylated CpG sites amongst the sites with ≥ 20% methylation in either control or follistatin treated groups, demonstrated a single CpG site at position -1374 that met the analysis threshold, this specific CpG site was hypomethylated in response to follistatin treatment (*P*<0.05; Fig 3E).

**Fig. 4:**
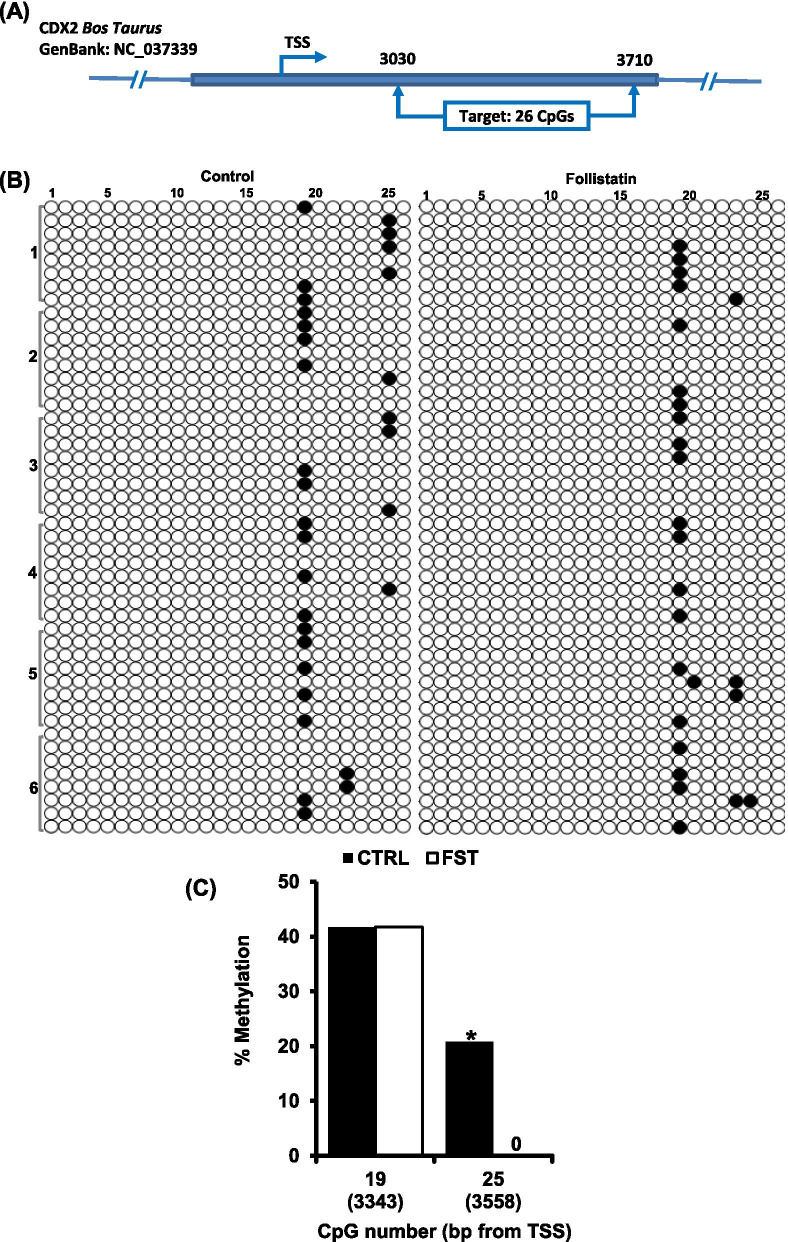
Follistatin treatment triggers changes in DNA methylation within intron 1 of *CDX2* gene in SCNT blastocysts. **A** *CDX2* gene map showing the genomic position of fragment I (+3030 to +3710). **B** Individual CpG methylation profile in fragment I, genomic DNA, from untreated control and follistatin treated groups (*n*= 6 replicates, *n*= 15 blastocysts/group), was subjected to bisulfite treatment followed by PCR amplification, cloning, transformation and finally colony PCR to select positive clones (*n*= 8 clones/replicate) for Sanger sequencing. In the methylation grid, rows represent the sequenced clones (Clones are arranged according to their replicates, replicate number are shown in the left) and columns represent CpG sites. Unmethylated CpG sites are represented by open circles while methylated CpG sites are represented by closed circle. **C** Individual CpG sites with ≥ 20% methylation, at least in one group. Values show the methylation percentage of each CpG site, and those values marked with an asterisk are significantly different (*P < 0.05)

For fragment P2, the target sequence was 431 bp in length (-126 to +305) and contained 41 CpG sites (Fig. 3B). The overall methylation rate was 4.4 ± 0.31 and 3.6 ±0.31% for control and follistatin treated embryos, respectively (Fig. 3D). Analysis of individual CpG methylation sites showed that only twelve CpG sites were methylated in 2.1-70.8% of the sequenced clones (Fig. 4D), with six CpG sites meeting the criteria for analysis (≥ 20 % methylation in either control or follistatin treated groups). Statistical analysis by Fisher’s exact test revealed that four CpG sites at positions -279, -163 and – 23, 122 were hypomethylated and two other sites at positions -243 and +20 were hypermethylated in response to follistatin supplementation (*P*<0.05; Fig. 3F).

### Follistatin treatment triggers changes in DNA methylation within intron 1 of *CDX2* gene in SCNT blastocysts

*CDX2* expression is regulated via multiple regulatory regions including the proximal promoter region and intron 1 [21, 22], therefore, a CpG rich fragment from intron 1 was selected for bisulfite sequencing analysis. The target sequence of the selected fragment (I) was 680 bp in length (+3030 to + 3710) and contained 26 CpG sites (Fig. 4A). The overall methylation rate was 2.5 ± 0.26 and 2.1 ± 0.32% for control and follistatin treated groups, respectively (Fig. 4B). Analysis of methylation of individual CpG sites showed that five CpG sites were methylated in 2.1 - 41.7% of the sequenced clones, with two CpG sites at positions +3343 and +3558 meeting the threshold for analysis, ≥ 20 % methylation in either control or follistatin treated groups (Fig. 4B). Further analysis by Fisher’s exact test revealed that CpG site at position +3558 was hypomethylated in response to follistatin treatment (*P*<0.05; Fig. 4C), whereas the CpG site at position +3343 was not differentially methylated; no statistical difference was observed between the two groups.

### Differentially methylated CpG sites are putative binding motifs for key transcription factors implicated in regulating *CDX2* expression

Transcription factor motif analysis combined with a literature search revealed several binding motifs in *CDX2* regulatory regions. These included key transcription factors implicated in *CDX2* transcriptional regulation. For example, in fragment P1, the differentially methylated CpG site (*n*=1) is putative binding site for Octamer-binding transcription factor 1 (OCT1) and Heart and neural crest derivatives expressed protein (HAND). In fragment P2, the hypomethylated CpG sites (*n*=4) contained putative binding sites for OCT1, Activating Enhancer-Binding Protein 2 family (AP2F), and p53 protein whereas the hypermethylated CpG sites (*n*=2) contained binding sites for Neuron-restrictive silencer factor (NRSF), a negative regulator of gene transcription. In fragment I of intron 1, there was a single hypomethylated CpG site in follistatin treated SCNT embryos which included a putative binding site for Krüppel-like transcription factor (KLF). Table 1 provides a full list of the transcription factors that are expected to bind to DM CpG sites in the *CDX2* promoter region and intron 1. Here, we focused only on TFs that are known to regulate *CDX2* expression in mouse embryos (OCT1, AP2F, KLF), and TFs that may have an indirect link to *CDX2* regulation (HAND, NRSF and P53). Collectively, these results indicate that follistatin treatment induces changes in DNA methylation at specific CpG sites within proximal promoter and intron 1 of *CDX2* in bovine SCNT blastocysts. These DM CpG sites contain binding motifs for key transcription factors implicated in *CDX2* regulation.

**Table 1:** Transcription factor motif analysis of methylated CpG sites effected by follistatin treatment

Fragment	Location (bp from TSS)	CpG methylation in response to FST	Putative transcription factors^a^
P1	-1374	↓	Oct1 RXRF HICF HAND HESF MYOD DEAF
P2	-279	↓	OCT1 STEM
	-243	↑	ZF5F E2FF
	-163	↓	AP2F ZFXY
	-23	↓	P53F
	20	↑	HNFP NRSF
	122	↓	WHNF ZTRE
I	3558	↓	KLFS ZF02 SAL BED EGR GLIF NDPK

*Abbreviations*: *bp* base pair, *TSS* transcription start site, *FST* follistatin

^a^Putative transcription factor binding sites that overlap differentially methylated CpG sites

## Discussion

A growing body of evidence supports an embryotrophic role of follistatin in IVF embryos [11–15, 27, 28]. Exogenous follistatin supplementation (10ng/ml) during IVC1, improved blastocyst rates of bovine SCNT embryos [9]. Here, we report that follistatin supplementation increases blastocyst rates as well as *CDX2* gene expression in bovine SCNT embryos. Previously, we demonstrated that follistatin treatment of bovine IVF embryos augments *CDX2* expression, at least in part, by modulation of the methylation status of *CDX2* gene. Follistatin induced hypomethylation of several CpG sites within the proximal promoter and intron 1 of *CDX2* gene in IVF blastocysts. These sites contain putative binding motifs for transcription factors implicated in regulation of *CDX2* expression in mouse embryos and trophoblast cells [16]. Therefore, we speculated that the effects of follistatin on *CDX2* expression may be conserved in SCNT embryos. To address this, we analyzed the methylation status of *CDX2* regulatory elements, proximal promoter, and intron 1 following the same procedures we utilized in IVF embryos [16]. Bisulfite sequencing analysis of selected fragments revealed that *CDX2* is generally hypomethylated which is in line with a previous study by Zhang and colleagues [29] who reported that *CXD2* is hypomethylated in bovine SCNT blastocysts.

Similar to our previous study in IVF embryos [16], follistatin treatment altered DNA methylation at specific CpG sites across the proximal promoter and intron 1 in SCNT embryos, however, some of the DM CpG sites are conserved in IVF and SCNT embryos, whereas others are exclusive for either IVF or SCNT embryos (Supplemental Table 1). In all cases, motif analysis revealed that DM sites contained putative motifs for transcription factors that are known to regulate *CDX2* expression including OCT transcription factors, AP2 family, and the KLF family. In Frag P1, follistatin treatment triggered hypomethylation of one CpG site -1374 in SCNT embryos compared to hypomethylation of 2 sites −1384 and−1283 and hypermethylation of one CpG site −1501 in IVF embryos. In Frag P2, Five DM CpG sites (-279, -243, -163, -23, 20) are conserved in SCNT and IVF embryos in addition to one DM CpG site in each group, -250 in IVF and 122 in SCNT embryos. In intron region (I) follistatin reduced methylation at one CpG site in SCNT embryos (3558) compared to five hypomethylated sites (3060, 3105, 3219, 3270, 3545) in IVF embryos.

In the present study, Motif analysis revealed two putative binding sites for OCT1 were found in fragment P1 (-1374) and P2 (-279), one binding site for AP2 family is in fragment P2 (-163) and one binding site for KLFS in fragment I (+3358). Follistatin also increased methylation at a specific CpG (20) site in fragment P2 that contained a putative binding site for the transcriptional co-repressor, neuron-restrictive silencing factor (NRSF). Notably, these results are consistent with our previous findings in IVF embryos [16] which supports the hypothesis that follistatin regulates *CDX2* expression via modulation of DNA methylation. Interestingly, follistatin treatment reduced methylation at putative binding sites for Heart and neural crest derivatives expressed protein (HAND) in fragment P1 and P53 protein in fragment P2. Although, to the best of our knowledge, there are no direct links between these transcription factors and *CDX2* regulation in embryos, it has been shown that HAND1 is essential for trophoblast differentiation in mice [30], while activation of P53 induced *Cdx2* expression in mouse embryonic stem cells [31].

## Conclusions

Results of the present study demonstrate that follistatin treatment during the first 72h of IVC enhances the developmental competence of bovine SCNT embryos and upregulates *CDX2* expression in blastocysts. The positive effects of follistatin on *CDX2* transcription are likely linked to alterations in DNA methylation within key regulatory regions of the *CDX2* gene. Collectively, these data provide additional insights into how follistatin treatment augments *CDX2* expression in bovine preimplantation embryos. Further epigenetic studies are required to elucidate the role of follistatin in bovine SCNT embryos.

## Supplementary Information

**Additional file 1: Supplemental Table 1**. Transcription factor motif analysis of methylated CpG sites effected by follistatin treatment in IVF and SCNT embryo

## Data Availability

All data generated or analyzed during this study are included in this published article

## Abbreviations

ATG: Translation initiation codon (methionine)
CDX2: Caudal type homeobox transcription factor 2
COCs: Cumulus oocytes complexes (COCs)
DM: Differential Methylation
FST: Follistatin
H: Hour
IVC: In Vitro Culture
IVF: In Vitro Fertilization
IVM: In Vitro Maturation
SCNT: Somatic Cell Nuclear Transfer
TE: Trophectoderm
TGA: Translation termination codon
TSS: Transcription Start Site
