# Supplementary material for: Follistatin supplementation induces changes in CDX2 CpG methylation and improves in vitro development of bovine SCNT preimplantation embryos
Source: Reprod Biol Endocrinol. 2021 Sep 13;19:141. doi: 10.1186/s12958-021-00829-7 (PMC8436481; doi:10.1186/s12958-021-00829-7)
Supplement: Supplementary file 1 — Additional file 1: Supplemental Table 1. Transcription factor motif analysis of methylated CpG sites effected by follistatin treatment in IVF and SCNT embryo [file 12958_2021_829_MOESM1_ESM.docx]

| **Fragment** | **Location**  **(bp from TSS)** | **CpG methylation**  **in response to FST** | **Affected Group** | **Putative transcription factors** |
| --- | --- | --- | --- | --- |
| P1 | −1501 | ↑ | IVF | EGRF, NRF1, ZF15, CTCF |
|  | −1384 | ↓ | IVF | NOLF, FXRE |
|  | -1374 | ↓ | SCNT | Oct1, RXRF, HICF, HAND, HESF, MYOD, DEAF |
|  | −1283 | ↓ | IVF | NRSF, STAF |
| P2 | -279 | ↓ | SCNT, IVF | OCT1, STEM |
|  | −250 | ↑ | IVF | E2F, NRSF, HESF, NRF1, ZF5F |
|  | -243 | ↑ | SCNT, IVF | ZF5F, E2FF |
|  | -163 | ↓ | SCNT, IVF | AP2F, ZFXY |
|  | -23 | ↓ | SCNT, IVF | P53F |
|  | 20 | ↑ | SCNT, IVF | HNFP, NRSF |
|  | 122 | ↓ | SCNT | WHNF, ZTRE |
| I | 3060 | ↓ | IVF | PAX2, SORY |
|  | 3105 | ↓ | IVF | HEAT, CTCF, AP2F |
|  | 3219 | ↓ | IVF | MYOD, GREF |
|  | 3270 | ↓ | IVF | KLFS |
|  | 3545 | ↓ | IVF | ZF02, SAL2, BEDF, EGRF, GLIF, KLFS, NDPK |
|  | 3558 | ↓ | SCNT | KLFS, ZF02, SAL, BED, EGR, GLIF, NDPK |

**Supplemental table 1: Transcription factor motif analysis of methylated CpG sites effected by follistatin treatment in IVF and SCNT embryo**

Abbreviations: FST, follistatin; TSS, transcriptional start site.

Putative transcription factor binding sites that overlap differentially methylated CpG sites.
